# Abnormal gait pattern emerges during curved trajectories in high-functioning Parkinsonian patients walking in line at normal speed

**DOI:** 10.1371/journal.pone.0197264

**Published:** 2018-05-11

**Authors:** Anna Maria Turcato, Marco Godi, Marica Giardini, Ilaria Arcolin, Antonio Nardone, Andrea Giordano, Marco Schieppati

**Affiliations:** 1 Division of Physical Medicine and Rehabilitation, ICS Maugeri SPA SB, Institute of Veruno, IRCCS, Veruno, Novara, Italy; 2 Centro Studi Attività Motorie, ICS Maugeri SPA SB, Institute of Pavia, IRCCS, Pavia, Italy; 3 Neurorehabilitation and Spinal Units, ICS Maugeri SPA SB, Institute of Pavia, IRCCS, Pavia, Italy; 4 Department of Clinical-Surgical, Diagnostic and Pediatric Sciences, University of Pavia, Pavia, Italy; 5 Unit of Bioengineering, ICS Maugeri SPA SB, Institute of Veruno, IRCCS, Veruno, Novara, Italy; 6 Department of Exercise & Sports Science, International University of Health, Exercise and Sports, LUNEX University, Differdange, Luxembourg; University of Illinois at Urbana-Champaign, UNITED STATES

## Abstract

**Background:**

Several patients with Parkinson´s disease (PD) can walk normally along straight trajectories, and impairment in their stride length and cadence may not be easily discernible. Do obvious abnormalities occur in these high-functioning patients when more challenging trajectories are travelled, such as circular paths, which normally implicate a graded modulation in the duration of the interlimb gait cycle phases?

**Methods:**

We compared a cohort of well-treated mildly to moderately affected PD patients to a group of age-matched healthy subjects (HS), by deliberately including HS spontaneously walking at the same speed of the patients with PD. All participants performed, in random order: linear and circular walking (clockwise and counter-clockwise) at self-selected speed. By means of pressure-sensitive insoles, we recorded walking speed, cadence, duration of single support, double support, swing phase, and stride time. Stride length-cadence relationships were built for linear and curved walking. Stride-to-stride variability of temporal gait parameters was also estimated.

**Results:**

Walking speed, cadence or stride length were not different between PD and HS during linear walking. Speed, cadence and stride length diminished during curved walking in both groups, stride length more in PD than HS. In PD compared to HS, the stride length-cadence relationship was altered during curved walking. Duration of the double-support phase was also increased during curved walking, as was variability of the single support, swing phase and double support phase.

**Conclusion:**

The spatio-temporal gait pattern and variability are significantly modified in well-treated, high-functioning patients with PD walking along circular trajectories, even when they exhibit no changes in speed in straight-line walking. The increased variability of the gait phases during curved walking is an identifying characteristic of PD. We discuss our findings in term of interplay between control of balance and of locomotor progression: the former is challenged by curved trajectories even in high-functioning patients, while the latter may not be critically affected.

## Introduction

During the activities of daily living, linear walking is frequently intermingled with turns and circular courses when moving within the environment, and more than 30% of the walking time is spent along curved trajectories [1]. Curved walking has been studied in terms of muscle activation [2,3], kinematics [4,3] and kinetics [3,5]. These studies have shown modifications in muscle activation, walking speed or kinematics during curvilinear trajectories [6,7,8,9]. Seemingly, the nervous system orchestrates both straight-ahead and curved walking by modifying the same basic coordination pattern [4,10]. However, due to the constraints of curved walking [4,11,12], curved path implies different biomechanical patterns [8]. Brain activation also differs [13], possibly because thalamo-cortical information transmission increases when balance tasks become more demanding [14].

Curvilinear trajectories represent a challenge for the elderly [15,16], because of the associated cognitive involvement, and bear a consistent risk of falling [17,18]. In patients with neurological disorders, more severe walking problems have been detected during curved than linear trajectories [7,19,20,21]. In particular, numerous studies have shown that patients with Parkinson’s disease (PD) show significant shortening of step length compared to healthy subjects during curvilinear with respect to linear trajectories [20,22]. Turning may also be associated with freezing and falling in PD [18,23].

Few studies to date have reported on spatio-temporal variables [20] and biomechanical strategies during curvilinear path in PD [24,25]. Those results were based on patient groups with mixed Hohen-Yahr stages and different mean age, and walking at different speeds, with or without the use of walking aids [26,27]. Other problems concerned differences in the control group characteristics. In particular, PD patients as a group usually walk at lower speed than healthy subjects (HS) [24,27,28,29], whereas patients in the early stages of the disease do not inevitably show major impairments in the gait pattern, including alterations of kinematic and kinetic variables profile, but rather balance problems at transients [30]. Sofuwa et al. [31] studied kinematic and kinetic variables of PD walking in line at lower speed than healthy subjects, and found a minor difference between groups only in ankle but not hip and knee kinematic and kinetic. Therefore, it is not clear whether the impairments found in patients walking along curved trajectories are expression of an altered control of the associated complex balancing strategy or are the accompaniment of reduced walking speed [32,33,34]. In the present study, we investigated changes in critical spatio-temporal gait parameters during curvilinear path in high-functioning PD patients. To our knowledge, this issue has never been addressed in mildly or moderately affected patients, especially in patients, whose gait speed is unaffected compared to that of normal subjects. This approach would be able to highlight gait abnormalities specifically connected with the production of curved walking.

Consequently, it seemed appropriate to compare the gait of PD to that of HS subjects spontaneously walking at the same speed on linear trajectories, as similar gait speeds can conceal potentially abnormal locomotor strategies. Are there significant changes in spatio-temporal variables of gait in PD patients, even when their walking speed closely matches that of healthy subjects? If so, does this occur when their effortless self-selected linear walking speed is within normal range? Does curved walking enhance gait abnormalities? Answers to these questions would give hints about the presence of initial deterioration in motor automaticity, which is a general feature in patients with PD [35], and advocate early pharmacological [36] or training [37] intervention, aimed at improving their performance under challenging gait conditions [38,39].

We hypothesized that, even when walking linearly at normal speed, alterations in several spatio-temporal gait variables would have been detectable in the PD participants, including duration of double-support phase [40] and stride-to-stride fluctuations [41,42,43,44], since gait variability is able to disclose subtle pathological gait impairments [6,41,45]. Both variables may be more telling than routine measures such as mean gait speed or gait phases duration [42,46]. We also hypothesized that, since walking along curved trajectories challenges dynamic balance [8,47] and the coordination between gait components [48,49,50], abnormalities in curved walking would be more severe in PD patients than in healthy subjects, including changes in the stride length-cadence relationship [51,52].

## Methods

### Participants

We recruited in the Laboratory of Posture and Movement of Veruno eighteen patients with mild to moderate Parkinson’s disease (PD), aged 71.4 ± 8.0 (mean ± standard deviation, SD) and with Hoehn-Yahr range 2–2.5. All patients were able to walk without aids in their daily activities, and were free from ankle or foot pathology or other conditions that could contribute to postural instability or movement dysfunction. They reported no falls in the last 6 months. Exclusion criteria were non-corrected visual deficits, postural hypotension or dizziness, orthopaedic or arthritic disease known to affect walking, other neurological disorders, absence of clinical asymmetry. Patients with cognitive impairment (Mini-Mental State Evaluation Test, MMSE <24 score [53]) were excluded. All patients were tested on a stable medication regimen for the four weeks before the study and were on-phase. From a large convenience sample of healthy subjects (HS), after careful collection of medical history and considering the same exclusion criteria as for the Parkinsonian patients, we recruited eighteen subjects who matched our patients for age and gender and for the self-selected walking speed along the linear trajectory. We did not exclude subjects exhibiting self-selected walking speed in the lower rank of published ranges of normality for an elderly population [54,55,56,57]. Table 1 shows the individual values of walking speed in the linear trajectory for the two cohorts.

**Table 1:** Match of gait speed during linear trajectories between Parkinson’s disease patients (PD) and healthy subjects (HS).

Participants	Gait speed (m/s)	
	PD	HS
**1**	0.72	0.69
**2**	0.91	0.87
**3**	0.98	1.02
**4**	1.05	1.07
**5**	1.06	1.14
**6**	1.12	1.15
**7**	1.18	1.16
**8**	1.22	1.18
**9**	1.22	1.18
**10**	1.23	1.20
**11**	1.25	1.24
**12**	1.30	1.31
**13**	1.33	1.33
**14**	1.33	1.34
**15**	1.45	1.50
**16**	1.52	1.52
**17**	1.53	1.60
**18**	1.58	1.63
**MEAN**	**1.22**	**1.23**
*SD*	*0*.*23*	*0*.*24*

The Institutional Central Ethics Committee (CEC) of Fondazione Salvatore Maugeri specifically approved the study and the consent procedures (approval number 806 CEC), and all participants signed the approved informed written consent.

### Procedures

HS and PD walked under three different conditions, in random order: linear walking (LIN) and curved (CUR) walking, clockwise (CW) and counter-clockwise (CCW), at self-selected speed. The linear path was a line of tiles in a corridor. The circle (1.2 m radius) was drawn with a tape fixed on the floor of a large room. Before data acquisition, each subject (both PD and HS) performed two practice trials for each condition to familiarize with the instrumentation and task. During LIN, subjects executed two consecutive 20 m walking trials [58], while during the CUR trajectory, they executed four equal length trials, twice for each CW and CCW motion. LIN and CUR walking trials were randomized. There was a total of 6 trials per participant. All participants were instructed to walk looking forward, head erect, and not to focus on the tiles or the tape unless for sporadic checks. All subjects understood the instruction well. All were naive to the experimental procedure and all succeeded in performing the tasks. The two initial and final cycles were automatically excluded from data acquisition by the software, because changes in spatial-temporal variables, albeit minimal [59], can occur at initiation and termination of gait [60,61]. Hence, in order to assess steady-state walking, each subject started walking 3 m before the first photocell and stopped 3 m after the second photocell. For the LIN trajectory, one photocell was placed at the beginning and one at the end of the 20-m pathway. For the CUR trajectories, photocells were set perpendicular to the path, in order to acquire the trial duration from the beginning of the first lap to the end of the third lap, encompassing an overall path of 20 m. The entire recording session lasted about one hour.

### Data collection

The Unified Parkinson’s Disease Rating Scale motor examination (UPDRS, part III) was administered to all patients by the same operator, who did not participate in patient recruitment and recording. That scale is considered the gold standard for quantifying signs and symptoms and measuring disease severity in PD [62], and the UPDRS part III contains 14 items that score motor impairments, balance and functional mobility [63]. UPDRS has demonstrated validity and good inter-rater reliability [64]. For all the patients, we scored lateralization of motor symptoms [65] (max value = 32), based on the criteria for identifying asymmetry reported by Uitti et al. [66].

### Gait analysis equipment

All participants were wearing shoes of the same type (Superga 2750 model, Italy). The Pedar-X System^®^ (Novel, Germany) was used to record, transduce and analyze foot-pressures in order to compute the temporal parameters of gait. The system connects to highly conforming, elastic pressure-sensitive insoles that cover the entire plantar surface of the foot, and consists of Bluetooth communication hardware, data acquisition and analysis software, and a proprietary calibration device. In this investigation, we exploited this system for step timing analysis. At the beginning of the session, the insoles corresponding to individual foot size were calibrated using the calibration device according to the manufacturer manual. Insoles were then placed inside the shoes and, before each trial, were reset to correct for pre-existing pressures. Data were collected at 100 Hz.

The Pedar-X system and the procedure adopted in this study have been previously shown to have good reliability for both LIN and CUR trajectories [5]. We considered the following temporal variables generated by the system for each gait cycle: duration of single support, double support, swing phase, and stride time. Cadence was also obtained by means of the Pedar software, which took note of the initial heel contact and toe off, and detected the duration of each gait phase for each foot. For each trajectory, stride length was estimated from gait speed (m/s) and cadence (steps/min), i.e. (speed/cadence * 120) [67], and was normalized to height (stride length/height) as suggested by [68,69].

For each trial, the mean value of the temporal variables of the right and left foot were separately computed (and referred to as IN and OUT foot with respect to the curved trajectory) and durations were converted to percentage of the total gait-cycle duration (%GC) for each leg. The double support represents the time period during which both feet are on the ground. Conventionally the double support includes two parts, called initial double support (in which weight is being shifted from controlateral to ipsilateral limb) and terminal double support (in which weight is being shifted from ipsilateral to controlateral limb). For the purpose of this analysis, during LIN, the initial double support of one limb and the terminal double support of the contralateral limb have been averaged to give the duration of the double support LIN. During CUR trajectory, though, initial and terminal durations may not correspond: hence, the double support IN was the double support duration recorded for the foot laying inside the trajectory, while the double support OUT was the double support duration for the foot laying outside the trajectory. The coefficient of variation (CV), which gives a measure of time variability, was calculated based on a range of 40–60 steps for each subject, and was analysed for each trajectory [42,44]. CV was computed for single support duration, double support duration and swing time for each individual, according to the formula: CV = standard deviation / mean × 100.

### Statistical analysis

For continuous (age, body weight and height) and categorical (sex) variables, unpaired Student’s t-test or chi-square test were used, respectively, to compare PD and HS group. We used parametric statistics for spatio-temporal variables of gait since they followed a normal distribution (p > 0.05, Shapiro-Wilk’s test for all variables) and their variances were homogeneous (Levene’s test, p > 0.05 for all variables). A regression analysis was conducted to assess the effect of disease asymmetry on speed and cadence and doube support phase when patients with PD walked towards the direction of the more or the less affected side. Since no asymmetry effect was observed, CW and CCW trials were not analysed as different conditions. Therefore, the values of each gait variable acquired during CW and CCW walking were pooled together for each participant for further analysis. However, feet were defined ‘internal’ (IN) or ‘external’ (OUT) depending on their position with respect to the circular trajectory and were considered separately for all the temporal variables of gait. Analysis of variance (ANOVA) assessed the differences between PD and HS in walking speed, cadence, step length and gait variability under the LIN and CUR walking conditions. For each variable, the effects of foot position with respect to the trajectory (LIN, OUT, IN) were compared by means of 2-way repeated-measures ANOVA (between the two groups HS, PD and within the three trajectory conditions LIN, IN, OUT). When ANOVA gave a significant result, Fisher´s LSD test was used for post-hoc comparisons. Further, the mean values of the principal spatio-temporal variables were presented with the 95% Confidence Intervals (CI), for a quick interpretation of the estimate [70].

The linear relationships between stride length LIN and stride length CUR, cadence LIN and cadence CUR, and stride length and cadence for both LIN and CUR were computed [71]. We calculated the intercept values for these relationships with an approach similar to Egerton et al. [72]. The y-axis (stride length) was moved to lie within the data range of cadence by subtracting the mean value of the x-axis (cadence) of both populations collapsed. In this way, differences in intercept values were not unduly biased by the position in the graph of a particular value. The same line of reasoning applied in the case of stride length when this was the independent variable, as in the relationship between stride length LIN and stride length CUR.

The plots of stride length and of cadence CUR vs LIN and the relationships stride length-cadence CUR vs LIN were analysed by regression analysis. Differences in slope and intercepts of the HS and PD regression lines were tested by the STATA command `regress´. The intercept value was computed on the data normalized to the mean value of both cohorts, in order to attribute a realistic meaning to the elevation of one compared to the other regression line in the range of the existing data points [72]. Alpha was set at 0.05 and beta at 0.20 to avoid the type I and II errors [73]. The statistical power value (1-beta), i.e. the probability of incurring in the type 2 error, was calculated for the primary spatiotemporal variables. The software programs STATISTICA (StatSoft, version 12.0) and STATA were used for the analyses.

## Results

Table 2 shows the clinical characteristics of the patients with PD and of the HS. Each group was balanced for gender (chi square, p > 0.5 within each group).

**Table 2:** Characteristics of participants.

	PD		HS		P
	mean	*SD*	mean	*SD*	
N. male; N. female	10;8		8;10		0.5
(% male)	(55.6)		(44.4)		0.70
Age (years)	71.4	*8*.*0*	72.7	*7*.*6*	0.61
Body Weight (kg)	72.6	*15*.*1*	68.1	*7*.*5*	0.26
Height (cm)	167.3	*5*.*9*	163.6	*6*.*4*	0.08
Duration of disease (years)	8.6	*3*.*1*			
Hohen and Yahr scale	2.1	(1.0–2.5)			
UPDRS III	18.1	(9–27)			
Asymmetry score	2.6	(0–6.0)			

P-values refers to the t-test analysis between Parkinson’s Disease patients (PD) and Healthy Subjects (HS). Range of values are shown in brackets. For difference in the gender proportion between groups, chi-square test was performed. SD, Standard Deviation; UPDRS III, Unified Parkinson’s Disease Rating Scale, motor section III. Asymmetry score was the difference between the higher minus the lower score.

Groups were similar for age, weight, height (unpaired t-test, p > 0.05) and gender proportion (chi square, p = 0.70). Mean disease duration was about 9 years, mean Hoehn-Yahr staging was 2.1 and mean UPDRS section III score was about 18. The Table also reports the mean asymmetry score. The score was calculated as the difference between the UPDRS scores of the two sides (higher minus lower score). Of note, for all patients except two, the score was higher in the side of disease onset. For the two incongruent patients, the asymmetry scored 1. All patients except one had asymmetry scores < 5, i.e. were not asymmetric according to Uitti et al. [66]. One patient scored 6.

### Spatio-temporal variables of gait

#### Speed, cadence and stride length

**Linear trajectory**. Table 3 shows the gait variables collected for the LIN trials. Owing to the inclusion criteria, walking speed during the linear trajectory was not different between PD and HS. Of note, neither cadence nor stride length (which were not part of the inclusion criteria) were different between PD and HS.

**Table 3:** Characteristics of gait during linear trajectory.

	PD		HS		P
Speed (m/s)	1.22 ± *0*.*23*	[1.11–1.33]	1.23 ± *0*.*24*	[1.11–1.35]	0.91
Cadence (steps/min)	118.6 ± *12*.*5*	[112.4–124.8]	115.0 ± *10*.*2*	[109.9–120.1]	0.34
Normalized stride length	0.75 ± *0*.*11*	[0.70–0.80]	0.78 ± *0*.*11*	[0.72–0.84]	0.39

Values are expressed as mean ± SD and CI in brackets. P-values refers to the t-test analysis between Parkinson’s Disease patients (PD) and Healthy Subjects (HS).

**Fig 1:**
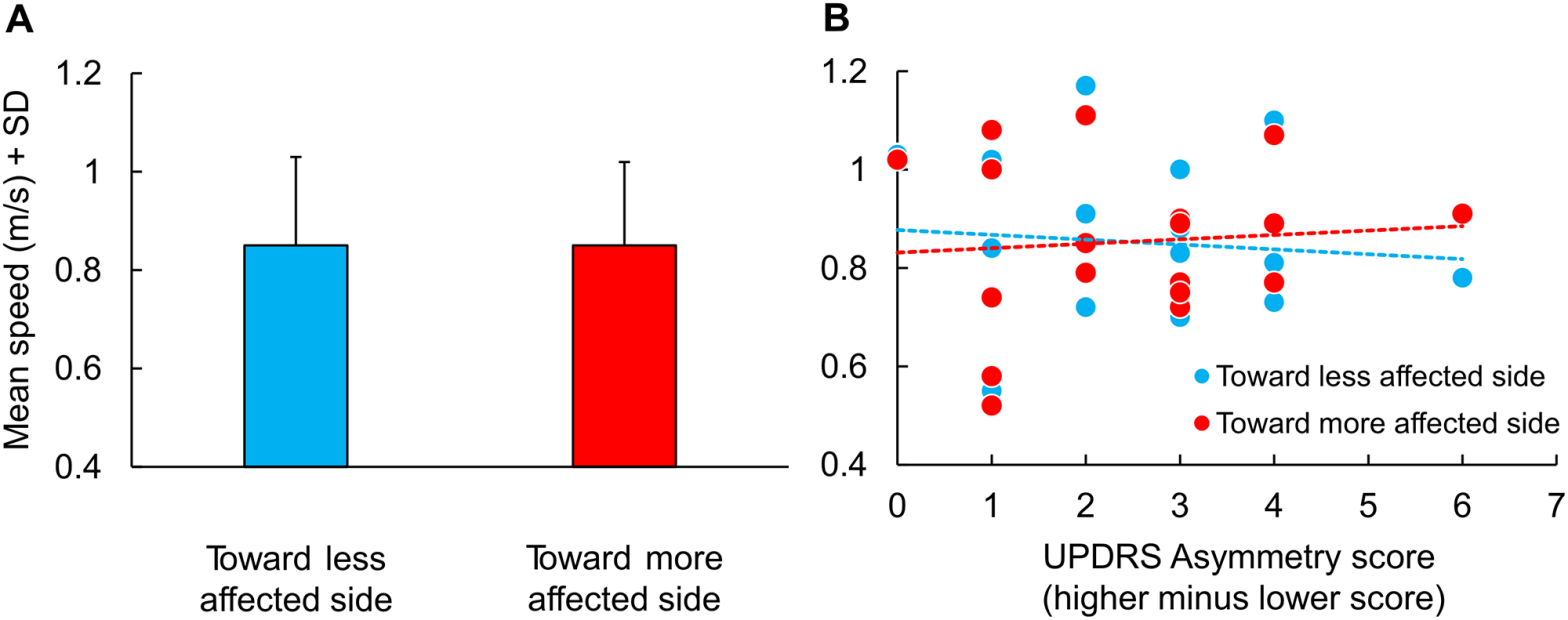
Mean speed of Parkinson’s Disease patients (PD) in turning toward the less and the more affected side and correlation between asymmetry score and mean speed. (A) Mean speed of Parkinson’s Disease patients (PD) in turning toward the less (blue column) and the more (red column) affected side. (B) Relationship between asymmetry score, computed from UPDRS, and mean speed used by PD for turning toward the less (blue dots) and the more (red dots) affected side. The slope of the best fit lines across each group are similar.

**Curved trajectory**. The small differences in the UPDRS score between the two sides did not allow to qualify the patients as asymmetric (except one). Patients´ speed data were preliminary grouped according to the body side with the prevalent severity of the disease, and the walking velocities were averaged depending on whether the direction of the trajectory was toward the more or the less affected side (i.e. the more affected side being internal or external to the trajectory, respectively). The analysis reported in Fig 1A shows that the side asymmetry (UPDRS scores, more affected minus less affected side) did not affect walking speed along the curved trajectories. No difference was found between the mean walking velocities when the patients travelled the curved trajectory towards the more or the less affected side (Student´s t-test, p = 1.0). Fig 1, part B, shows the speed values for each patient plotted against their own asymmetry score, separately for the direction of the curved trajectories. There was no significant effect of asymmetry on walking speed, and the regression lines were almost flat (y = -0.01x + 0.88, R^2^ = 0.01, p = 0.74 and y = 0.01x + 0.83, R^2^ = 0.01, p = 0.75, for motion toward the less and the more affected side, respectively). The points pertaining to the patient with an asyimmetry score of 6 were very close to the mean values for both directions of the curved trajectories. Further, there were no differences in cadence between turning in the direction of the more (109.3 steps/min ± 12.3) or of the less affected side to (109.2 ± 13.4) (t-test, p = 0.91).

**Fig 2:**
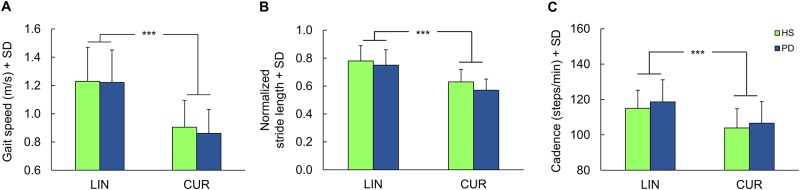
Gait variables during linear and curved walking in Parkinson’s Disease patients (PD) and healthy subjects (HS). (A) Gait speed. (B) Normalized stride length. (C) Cadence. ***, P < 0.0005.

Hence, CW and CCW trials were not analysed as different conditions but pooled together, in order to highlight any effect of the disease on the performance of curved walking (strongly dependent on the constraints represented by the curved trajectory). Fig 2 shows that the type of the trajectory affected gait variables in *both* HS and PD groups. Speed diminished by about 30% for PD and 27% for HS during curved (CUR) with respect to LIN walking (Fig 2A). Stride length diminished during CUR with respect to LIN in both groups, more so in PD (about 24%) than HS (19%) (Fig 2B). Cadence diminished during CUR with respect to LIN in both groups as well: it was reduced by about 11% in PD and 10% in HS (Fig 2C).

ANOVAs showed that walking speed (F = 47.8, df = 1,68, p < 0.0001), cadence (F = 18.3, df 1,68, p < 0.0001) and stride length (F = 48.2, df = 1,68, p < 0.0001) were significantly different during CUR compared to LIN trajectories. There were no between-group significant differences for speed (F = 0.15, df = 1,68, p = 0.70) and cadence (F = 1.36, df = 1,68, p = 0.25). There was a marginally significant difference for stride length, shorter in PD (F = 3.97, df = 1,68, p = 0.05).

No interactions between groups and trajectories were found in the analysis pertaining to each of these three variables, indicating that broadly similar changes in speed, stride length and cadence were induced by CUR walking in both HS and PD patients. Post-hoc tests showed no differences in PD patients compared to HS during CUR walking for speed and cadence (p = 0.53 and p = 0.48, respectively). The marginal difference for stride length, shorter in PD, was due to the relatively shorter stride length compared to HS during CUR walking only (post-hoc PD vs HS; LIN, p = 0.33; CUR, p = 0.07).

**Fig 3:**
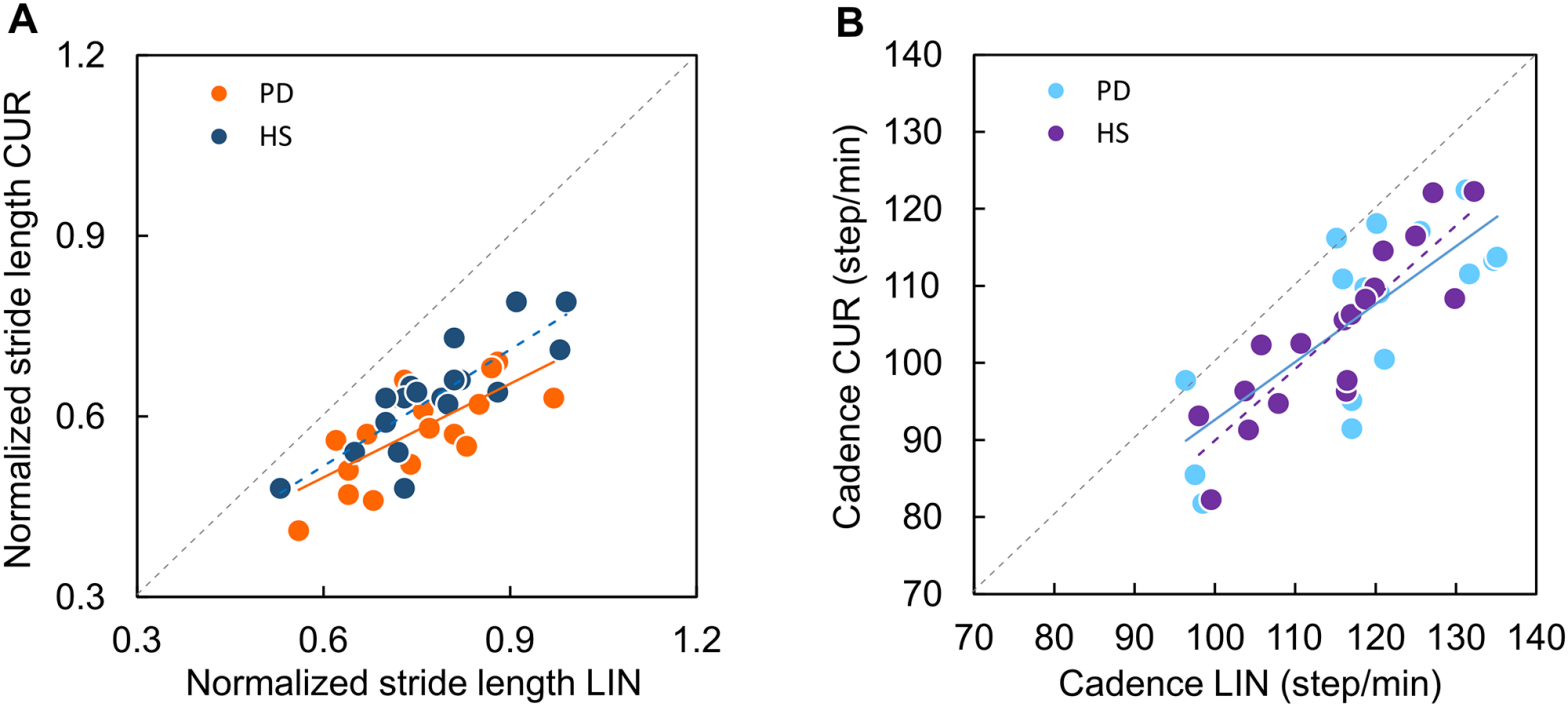
Relationship between gait variables during linear and curved trajectories in HS and PD. (A) Relationship between normalized stride length during linear (LIN) and curved (CUR) trajectories in HS (blue dots) and PD (orange dots). The slopes of the best fit line across each group are similar but HS have a longer stride length in relation to cadence during CUR. (B) Relationship between cadence during linear (LIN) and curved (CUR) trajectories in HS (violet dots) and PD (sky-blue dots). The slopes of the best fit line across each group are similar.

Fig 3A shows that stride length clearly diminished in both HS and PD patients during CUR with respect to LIN walking (the identity line, CUR = LIN, is the dotted diagonal). The slope of the regression line fitted to the scatterplot of stride length CUR vs LIN was significant both in HS (blue circles, y = 0.63x + 0.14, R^2^ = 0.68, p < 0.0001) and in PD (Fig 3A, orange circles, y = 0.53x + 0.18, R^2^ = 0.50, p < 0.005). The slopes were not different between HS and PD (t-test on the regression slopes, t = 0.32, p > 0.74). Thus, subjects exhibiting a short stride in LIN had a short stride in CUR as well, and those with long strides had long strides under both conditions. The elevation of the CUR vs LIN regression lines of HS and PD patients was significantly greater (t-test = 2.14, p = 0.04), in keeping with most of data points of the PD patients laying below those of the HS. This corresponds to the reduction in the mean stride length observed in PD patients compared to HS during CUR walking mentioned above.

Fig 3B shows that cadence also diminished during CUR walking with respect to LIN, to the same extent in PD and HS. The slope of the lines best fitting the scatterplot of cadence CUR versus cadence LIN was significant both in HS (purple circles, y = 0.93x -2.95, R^2^ = 0.76, p < 0.0001) and in PD patients (light blue circles, y = 0.75x +17.57, R^2^ = 0.58, p < 0.0005). No difference was found in the slopes between groups (t = 0.09, p = 0.9). The elevation of the regression lines of HS and PD patients was not significant, either (t = 0.84, p = 0.41), meaning that cadence did not disproportionately diminish during CUR in PD patients, regardless of the value of their LIN cadence.

### Relationship between cadence and stride length

Because of the similar walking speed between PD patients and HS along the CUR trajectories, we examined the plots of stride length versus cadence in both groups, under both walking conditions.

**Fig 4:**
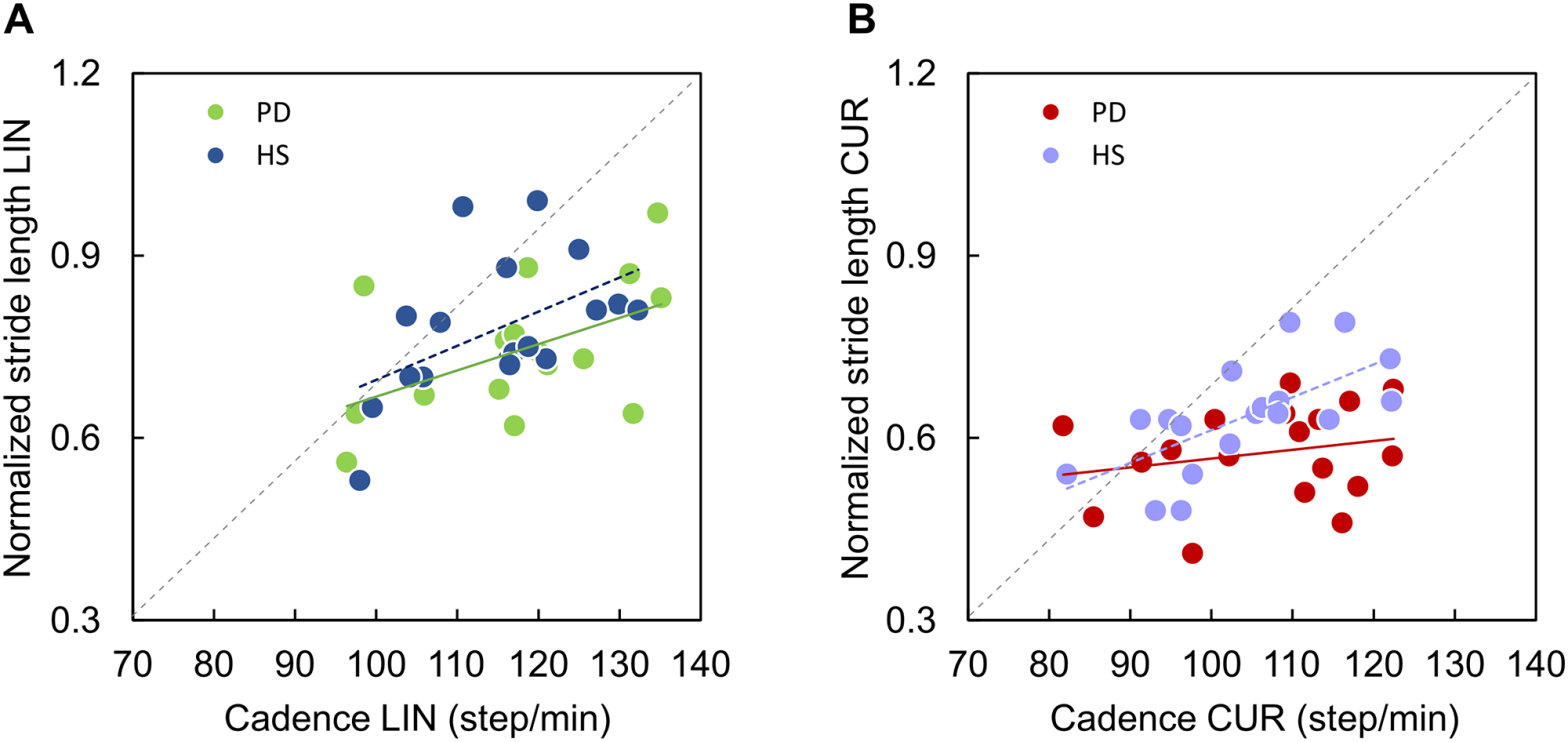
Relationship between normalized stride length and cadence in HS and PD. (A) Relationship between normalized stride length and cadence during linear (LIN) trajectory in HS (blue dots) and PD (green dots). (B) Relationship between normalized stride length and cadence during curved (CUR) trajectory in HS (violet dots) and PD (bordeaux dots).

Fig 4A shows that the stride length-cadence relationships during LIN trajectory were similar in PD patients and HS. The relationship between cadence and stride length was y = 0.004x + 0.23, R^2^ = 0.26, p = 0.03, and y = 0.006x + 0.14, R^2^ = 0.25, p = 0.03, for PD and HS, respectively. Slope (t = 0.43, p = 0.67) and elevation (t = 0.29, p = 0.14) of the regressions for PD and HS were not significantly different. The same analysis made for the CUR trajectory showed that the slope of the stride length-cadence relationship was significant only in HS (Fig 4B: brown circles, y = 0.005x + 0.07, R^2^ = 0.68, p < 0.002), where subjects with high cadence had the highest stride length. For the PD group, the relationship between stride length and cadence was almost flat during curved walking (violet circles, y = 0.001x + 0.41, R^2^ = 0.05, p = 0.36) (Fig 3B), indicating a reduction of stride length in PD patients, even in those exhibiting a fair cadence. Therefore, the two regressions showed a significantly different slope in HS and PD during curved walking (t-test on the regression slope, t = 3.27, p < 0.01). The intercept of the regression lines was also significantly different (t-test = -1.5, p = 0.01).

### Duration of the gait phases

The mean values of the duration of the gait phases are reported in Table 4.

**Table 4:** Mean duration of gait phases (ms).

Walking conditions	Stride time (ms)		Single Support (ms)		Double Support (ms)		Swing phase (ms)	
	HS	PD	HS	PD	HS	PD	HS	PD
**LIN**	1041	1048	374	367	149	159	379	372
*SD*	*85*	*128*	*32*	*36*	*17*	*41*	*33*	*37*
**IN**	1168	1148	412	383	183	203	403	373
*SD*	*125*	*160*	*39*	*50*	*30*	*51*	*44*	*52*
**OUT**	1167	1147	399	368	179	197	417	389
*SD*	*124*	*159*	*43*	*50*	*30*	*52*	*39*	*51*

LIN, linear trajectory; IN, foot inside the curved trajectory; OUT, foot outside the curved trajectory; Parkinson’s Disease patients (PD) and Healthy Subjects (HS).

**Stride time**: There was an equivalence of the duration of the stride time across subjects during LIN walking. Mean stride time was about 20 ms shorter in PD with respect to HS for CUR walking (for both the inner and the outer leg). Differences between PD and HS were also found in the duration of the double support phase, which was more prolonged in the former than in the latter group by about 10 ms (LIN) and 20 ms (CUR). Coherently, the single support duration was shorter in PD than HS across conditions and legs. Since the stride times were similar between PD patients and HS, we performed the analysis on the duration of the gait phases expressed in percent of gait cycle duration (%GC).

**Fig 5:**
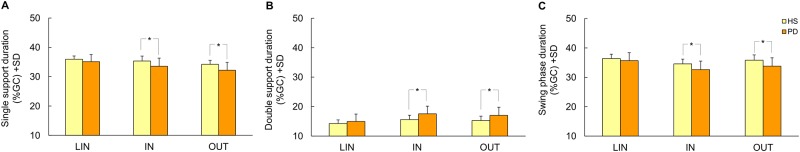
Temporal variables of gait during linear (LIN) and curved walking in PD and HS. **D**uring curved walking the foot inside (IN) and outside (OUT) the trajectory is considered separately. *, P<0.05. (A) Single support duration expressed in % of Gait Cycle. (B) Double support duration. (C) Swing phase duration.

**Single support phase**: The minor asymmetries of the patients with PD had no effect on the duration of their single support phase when walking along the curved trajectories in the direction of the more or the less affected side (t-test, p = 0.67). Fig 5A shows the mean duration of the single support during LIN and CUR (IN and OUT) walking. For PD, it was 35.1 ± 2.4 (CI varying from 33.9 to 36.3), 33.6 ± 2.8 (CI 32.3–34.9), and 32.2 ± 2.7 (CI 31.0–33.4), during LIN, IN and OUT conditions, respectively. The mean values of the single support durations for HS were 36.0 ± 1.1 (CI 35.4–36.5), 35.4 ± 1.7 (CI 34.6–36.1), and 34.2 ± 1.4 (CI 33.6–34.8) for LIN, IN and OUT, respectively.

Two-way ANOVA showed that the differences in single support duration were significant between groups (ANOVA, F = 5.36, df 1,68, p < 0.05) and trajectories (ANOVA, F = 71.54, df 2,68, p < 0.0005). The interaction was also significant (ANOVA, F = 8.18, df 2,68, p < 0.005), because the single support was shorter in PD than HS under CUR walking condition. Post-hoc test showed that both groups reduced the single support duration of the foot inside (IN) and outside the trajectory (OUT) compared to LIN walking (post-hoc, IN vs LIN p = 0.03 and p < 0.001, for PD and HS respectively; OUT vs LIN p < 0.0001, both for PD and HS). PD patients reduced the duration of single support during CUR walking to a larger extent than HS (post-hoc, p < 0.01 between PD and HS, for both foot IN and OUT).

**Fig 6:**
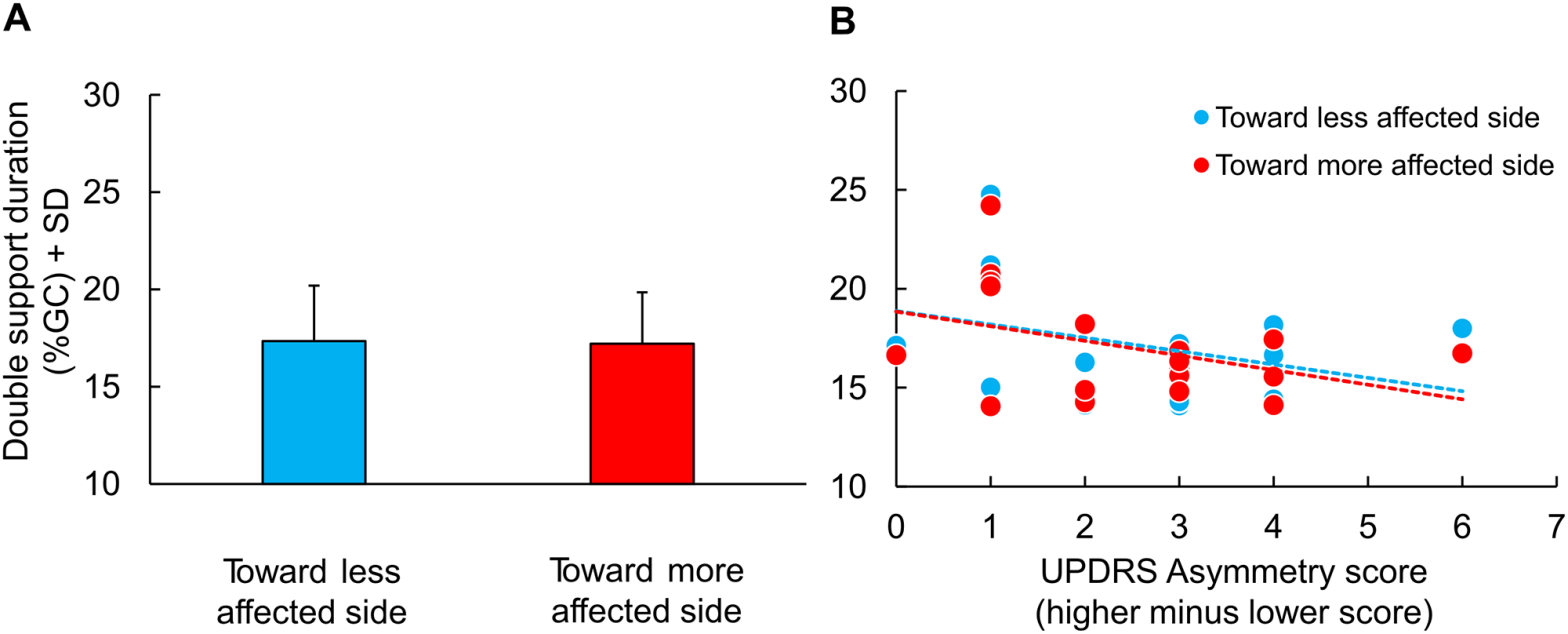
Mean double support phase in % of gait cycle (GC) of Parkinson’s Disease patients (PD) in turning toward the less and the more affected side and correlation between asymmetry score and mean double support phase. (A) Mean double support phase in %GC of PD turning toward the less (blue column) and the more (red column) affected side. (B) Relationship between asymmetry score, computed from UPDRS, and mean double support phase in PD turning toward the less (blue dots) and the more (red dots) affected side. The slope of the best fit lines across each group are similar.

**Double support phase**: Fig 6A shows that the minor asymmetries detected in our patient group did not affect the duration of the double support when walking along the curved trajectories in the direction of the more or the less affected side (t-test, p = 0.56). Part B shows that the duration of the double support was not dependent on the UPDRS asymmetry score across patients and curved-walking directions (y = -0.68x + 18.87, R^2^ = 0.12, p = 0.15 and y = -0.74x + 18.85, R^2^ = 0.16, p = 0.10, for walking toward the less and the more affected side, respectively).

Fig 5B shows the duration of the double support phase in %GC. The duration for HS was 14.3 ± 1.2 (CI varying from 13.7 to 14.9), 15.6 ± 1.5 (CI 14.9–16.3), 15.3 ± 1.5 (CI 14.6–16.0) for LIN, IN and OUT respectively. For PD, it was 15.0 ± 2.5 (CI 13.7–16.2), and 17.5 ± 2.6 (CI 16.3–18.7), 17.0 ± 2.8 (CI 15.7–18.3), during LIN, IN and OUT conditions, respectively, featuring a consistent relative increase of the double support phase during CUR walking compared to HS. Two-way ANOVA showed significant differences in double support duration (in %GC) between groups (ANOVA, F = 4.71, df 1,68, p < 0.05) and between trajectories (ANOVA, F = 72.65, df 2,68, p < 0.0005) (Fig 5B). The interaction was significant (ANOVA, F = 8.18, df 2,68, p < 0.001). Post-hoc test showed that duration was not different during LIN walking between groups (p = 0.34). However, even if both groups decreased the duration of swing-phase during CUR walking, PD reduced the duration of this phase more than HS. This was true for both the foot inside and the foot outside the trajectory (PD vs. HS, foot IN, p = 0.008; foot OUT, p = 0.02).

**Swing phase**: Swing-phase duration (in %GC) in PD during LIN, foot IN and foot OUT was 35.6 ± 2.7 (CI 34.3–37.0), 32.6 ± 2.9 (CI 31.2–34.1) and 33.5 ± 2.9 (CI 32.2–34.9), respectively. The corresponding values in HS were 36.4 ± 1.5 (CI 35.6–37.1), 34.6 ± 1.6 (CI 33.8–35.4) and 35.8 ± 1.8 (CI 35.0–36.7), respectively. Two-way ANOVA showed significant differences in swing-phase duration (Fig 5C) between groups (ANOVA, F = 5.75, df 1,68, p < 0.05) and between trajectories (ANOVA, F = 35.07, df 2,68, p < 0.0005). The interaction was significant (ANOVA, F = 3.99, df 2,68, p < 0.05). Post-hoc test showed that, even if both groups decreased the duration of swing-phase during curved walking, patients with PD reduced the duration of this phase more than HS. This was true both for the foot inside and for the foot outside the trajectory (PD vs. HS, foot IN, p = 0.01; foot OUT, p = 0.004).

The statistical power (1 − β) for the differences in duration of the gait phases between PD and HS are reported in Table 5 (left column). Significant power values (for both foot IN and foot OUT) are highlighed in bold. It appears that the differences in duration of all phases bear a strong statistical power for the curved trajectories, the linear trajectory variables bearing the least statistical power.

**Table 5:** Statistical power (1 − β) for the differences in duration and variability of the gait phases.

	Phase duration			Variability of duration		
	LIN	IN	OUT	LIN	IN	OUT
**Single Support**	0.48	**0.85**	**0.91**	0.10	0.75	**0.85**
**Double Support**	0.34	**0.96**	**0.91**	0.11	0.21	0.33
**Swing phase**	0.33	**0.83**	**0.89**	0.13	**0.96**	**0.99**

LIN, linear trajectory; IN, foot inside the curved trajectory; OUT, foot outside the curved trajectory. Significant power values are reported in bold.

### Coefficient of variability

**Fig 7:**
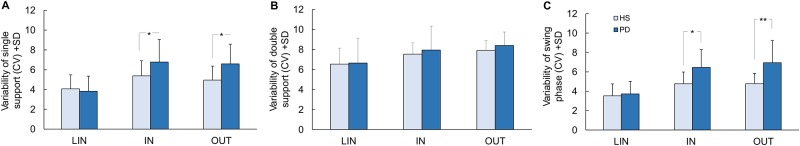
Coefficient of variability (CV) of temporal gait variables during linear (LIN) and curved walking in PD and HS. During curved walking the foot inside (IN) and outside (OUT) the trajectory is considered separately. *, P<0.05 **; P<0.005. (A) CV of single support duration. (B) CV of double support duration. (C) CV of swing phase duration.

Fig 7 shows the variability of the gait phases (in percent of the gait cycle) across groups and walking conditions. The coefficient of ariability (CV) was larger for CUR (for both IN and OUT feet) than LIN walking, and in the former case far larger in patients with PD than HS. There was no differential increase in variability of the phase durations between the inner and the outer leg. Interestingly, this increment in variability was much larger for the dynamic swing phase (or, reciprocally, for the single support phase) than for the double support.

**Variability of single support duration**: The mean coefficient of variability of the duration of the single support during LIN was 3.8 ± 1.5 (CI varying from 3.1 to 4.6) and 4.1 ± 1.4 (CI 3.4–4.8), in PD and HS, respectively (Fig 7A). During the CUR trajectories, CV was 6.8 ± 2.3 (CI 5.7–7.9) for the foot IN and 6.6 ± 2.0 (CI 5.7–7.5) for the foot OUT in PD, while in HS was 5.4 ± 1.5 (CI 4.6–6.1) for foot IN and 5.0 ± 1.4 (CI 4.3–5.6) for foot OUT. The mean CV of single support duration was significantly higher in PD than HS (two-way ANOVA, F = 3.93, df 1,68, p < 0.05). There was an effect of the trajectory (ANOVA, F = 32.81, df 2,68, p < 0.0005) and an interaction between groups and trajectories (ANOVA, F = 6.48, df 2,68, p < 0.005). Post-hoc showed no differences in variability between the two groups during LIN (p = 0.67). However, both groups significantly increased the variability of the single support duration during CUR walking (IN vs LIN, p < 0.0001 and p = 0.002, for PD and HS respectively; OUT vs LIN, p < 0.0001 and p = 0.03, for PD and HS respectively). Moreover, both CVs of foot IN and OUT were higher in PD than HS (foot OUT PD vs HS, p = 0.006; foot IN PD vs HS, p = 0.02).

**Variability of double support duration**: The CV of the double support duration during LIN was 6.6 ± 2.6 (CI 5.4–7.9) and 6.5 ± 1.6 (CI 5.7–7.4), in PD and HS respectively (Fig 7B). During CUR trajectory, variability was 7.9 ± 2.5 (CI 6.7–9.1) for the foot IN and 8.4 ± 1.3 (CI 7.8–9.0) for the foot OUT in PD, while in HS it was 7.5 ± 1.1 (CI 7.0–8.0) for foot IN and 7.9 ± 1.0 (CI 7.4–8.4) for foot OUT. The mean CV of double support duration was not different in PD and HS (ANOVA, F = 0.05, df 1,68, p = 0.82). However, there was an effect of trajectory (ANOVA, F = 8.18, df 2,68, p < 0.001), variability being higher in the foot IN and foot OUT with respect to LIN in both groups. There was no interaction between groups and trajectories (ANOVA, F = 0.61, df 2,68, p = 0.54).

**Variability of swing-phase duration**: The CV of the swing-phase duration matched that of the single support phase. It was 3.7 ± 1.3 (CI 3.1–4.4) and 3.5 ± 1.2 (CI 2.9–4.1) during LIN, respectively in PD and HS (Fig 7C). During CUR trajectory, CV was 6.5 ± 1.8 (CI 5.6–7.3) for the foot IN and 6.9 ± 2.3 (CI 5.8–8.1) for the foot OUT in PD. CV was 4.8 ± 1.2 (CI 4.2–5.3) for foot IN and 4.8 ± 1.1 (CI 4.3–5.3) for foot OUT in HS. Two-way ANOVA showed significant differences in variability of swing-phase between groups (ANOVA, F = 9.01, df 1,68, p < 0.005) and between trajectories (ANOVA, F = 61.94, df 2,68, p < 0.0005). The interaction was significant (ANOVA, F = 10.91, df 2,68, p < 0.0005). Both groups significantly increased the variability of the single support duration during CUR walking but to a larger extent PD patients (foot OUT PD vs HS p < 0.0001; foot IN PD vs HS p < 0.005).

The statistical power for the differences in variability between PD and HS are reported in Table 5, right part. Note that the variability of the double support LIN bears the least statistical power, while the variability of the swing phase for both feet bears a higher statistical power.

## Discussion

Our results confirm the hypothesis that the spatio-temporal pattern of gait is modified in patients with PD with respect to healthy subjects (HS) while walking along curved trajectories, even when the patients exhibited no difference in walking speed along a linear path compared to the HS. Interestingly, we found no significant differences in walking speed and gait cadence along the curved trajectory, either, between the patients and the HS, even if equal speed during curved walking was not a pre-condition for patient selection. However, stride length during curved walking diminished slightly more in patients with PD than in HS. In addition, modifications in gait timing during curved walking in PD compared to HS consisted in reduced single support and swing phase duration, increased double support duration, increased variability of the single support, and swing phase duration.

Most of these patients with PD had H-Y scores of 2. Therefore, they were not severely affected patients, but were not free from motor problems, since their mean UPDRS motor score was 18. These patients walked without apparent effort at the same speed as their healthy peers. They were not deliberately encouraged to walk at a higher than their preferred speed. It cannot be excluded that some of them tried to perform at the best of their possibilities just because they were tested in a laboratory. However, they were not forced to walk fast, or were ever told that their performance would have been paired to that of the HS. Moreover, they were medicated and were on-phase. They did not have freezing of gait either during the experimental trials or in their clinical history. Of note, all the patients were rated as non-asymmetric (the side difference in the UPDRS score was < 5), except one for whom the difference was 6. This patient did not perform differently than his peers, as per all recorded gait variables. Moreover, there was no effect of the asymmetry scores on speed and double support phase when these patients walked along the circular trajectories toward the more or the less affected side.

### Walking speed during linear walking: High-functioning patients show no difference from matched healthy subjects

Contrary to other studied cohorts [74,75], these patients with PD and the ad-hoc selected HS had equal mean walking speeds during the linear trajectory. Cadence and stride length were not significantly different between groups, either, even if we noted a slightly shorter stride length and a slightly higher cadence in PD, as in Egerton et al. [76]. The similarity of PD and HS in these main variables during linear walking indicates good management of the basic mechanisms of walking. It is conceivable that the central pattern generator (CPG), which controls not only cadence but also the level of recruitment of muscle strength, and modulates the feedback from receptors [77], was essentially normal in these patients. Not alternatively, mechanisms of compensation, which are known to occur in Parkinson´s disease [78], would have taken place, and succeeded in appropriately modulating the rhythmic activity of the central pattern generator during steady-state linear walking [79]. Apparently, these patients would also have a satisfactory control onto the dynamic distance between the centre of mass of the body and the centre of foot pressure, which sets stride length and progression [80,81,82]. Overall, linear walking showed no abnormalities in the principal spatio-temporal gait variables and did not seem to represent a critical task in these high-functioning PD patients.

### Walking speed during curved walking does not separate high-functioning PD patients from healthy subjects

Walking along curved trajectories requires seamless interlimb coordination, adapted foot placement and fine control of medio-lateral body inclination [4,9,11,83,84]. Here, curved walking significantly decreased walking speed, stride-length and cadence both in HS and in patients with PD. Yet, these patients, deliberately matched to the HS based on their spontaneous speed during linear walking, showed no major differences in gait speed and cadence even during curved walking compared to HS. Of note, gait speed was not affected by the minor side asymmetry in the individual patients. The gait speed of the patients with the largest asymmetry expressed as difference in UPDRS scores was not separable from that of the group as a whole. The mean walking speed of the PD group was slightly lower and cadence was slightly higher than in the HS group, but these differences did not reach significance. Conversely, the mean stride length was just significantly shorter in PD than HS. The difference was small, however not unexpected for this patient cohort. The finding of relatively limited differences in walking speed between PD and HS during both linear and curved walking insinuates that curved walking did not imply any major specific abnormality in these high-functioning patients. Abnormalities might have been expected based on reports about the cognitive cost of walking or curved walking in PD [85,86,87]. These cohorts, however, had no cognitive impairment, as assessed by the Mini-Mental State Evaluation Test, and curved walking is more likely to be impaired when a cognitive double task is performed [88,89], a condition not tested in the present investigation.

### The stride length-cadence relationship emphasizes the reduction in stride length during curved walking in PD patients

We are not aware of published studies addressing the stride length-cadence relationship during steady-state curved walking, either in PD or HS. Therefore, we were interested in plotting the relationship between these two basic gait characteristics, in order to ascertain whether it was sensitive to curved walking in separating these patients with PD from the HS. During linear walking, the stride length-cadence relationships of HS and PD were similar to those reported by Egerton et al. [76] and Danoudis and Jansek [90]. The lines pertaining to the PD patients walking in line showed no significant changes in slope or elevation compared to HS. The similar slope indicates that there is no ceiling or floor effect for these variables across patients, at least during linear walking at self-selected speed [91]. This enforces the connotation of the stride length-cadence relationship as a basic coordination mechanisms and fundamental determinant of gait [92]. Also the cadence data points were in the same range in both populations.

The same relationship stride length-cadence drawn for curved walking showed that the data points of as many as 15 out of 18 patients were placed below the regression line drawn through the data points of the HS during curved walking. Interestingly, the slope was not significant in the case of PD, possibly indicating a ceiling effect for stride length across patients during curved walking. This alteration in the stride length-cadence relationship would reflect impaired coordination mechanisms, with disruption to scaling of stride length [76]. Therefore, during curved walking within a similar cadence range, some patients had a shorter stride length even at relatively high cadences, while the patients walking with a low cadence did not congruently decrease stride length. Further, the patients´ data points were more scattered than those of HS, featuring a large intragroup variability in this relationship. There might be confounding factors possibly connected with potentially different degrees of efficacy of the medication [93], or affective disturbances in this cohort [94] or initial signs of higher level gait disorders [95], which were not controlled for in this study. But the above confounding factors should have affected linear walking as well. Not unlikely, the short stride length of curved walking would rather reflect a specific, circumscribed disorder in postural control in these Parkinsonian patients, as discussed below.

### The duration of the gait phases is affected by curved walking in PD patients

Curved walking entails definite reorganization within the gait cycle. Changes in the duration of the single and double support phase and the respective differences between the inner and outer foot were observed in the present healthy aged subjects, and repeated those observed previously in younger persons walking along curved trajectories [6,10,11,15]. In the high-functioning PD patients studied here, the changes in duration of the single support and of the swing phase broadly replicated the changes from straight to curved walking observed in the HS. This suggests that the overall kinematic changes induced by curved walking in normal subjects [8,11] are preserved in PD patients. However, during curved walking, the durations of single support and swing phase were significantly shorter in PD than HS. These shorter durations were obviously accompanied by significant increases in the duration of the double support phase in PD patients, again only during curved but not linear walking. Walking speed *per se* would not have changed the relative duration of the gait phases, since all our patients and subjects walked under both linear and curved conditions at speeds higher than 0.7 m/s [96]. As suggested by several studies, the increased double support duration and the reduced single support duration in PD during curved walking would be strategies adopted by the patients to achieve superior postural stability during turning [19,97]. Interestingly, the double support duration during curved walking in this cohort of patients with absence of clinical asymmetry was not related to the minor side asymmetry spotted by the UPDRS scores. This observation, admittedly based on a small sample of patients, is not against the suggestion that the prevalent challenge of curved walking is posed by its inherent mechanical constraints than by any uneven effect of the disease. It will be critical indeed to investigate whether side disparity of motor signs, including deficits in trunk control, differently affects curved walking depending on motion direction. The increase in the double support phase is also common to other conditions, like peripheral neuropathy (particularly when this leads to static balance problems), in spite of a near normal gait [98]. Overall, it seems safe to state that these changes in the duration of the gait phases in these high-functioning PD patients are connected to their postural unsteadiness rather than to problems in the organization or implementation of the command for walking.

### Variability of the gait phases

Variability of spatial temporal gait parameters is considered a reliable index of inadequate walking, and has been shown to differ between fallers and non-fallers. Variability of stance or swing phase duration has been repeatedly associated with the risk of falling in different populations [99,100,101,102]. Variability of temporal gait parameters is greater in patients with Parkinson´s disease than in normal subjects [103,104].

We found a significantly larger variability of the temporal gait parameters in PD patients during curved but not linear walking, over and above the increased variability exhibited by HS [105]. We have no other explanation for this increase in variability but an abnormal coordination between posture and gait in these patients, possibly due to loss or decline in automaticity [36]. Variability of double support was also slightly increased in PD patients during curved walking compared to HS (though not significantly so). In absolute terms, however, the variability of the double-support phase was less important in PD patients than the variability in the other gait phases. Double support is a critical period, during which body balance control at the foot acceptance phase is checked in the face of the accountability of the last part of the single stance phase of the opposite leg, in order to produce the next step. It is considered an important indicator of abnormal balance control in both healthy subjects and patients with PD [106]. It might be viewed as the period during which balance control mechanisms intrude into, and allow, the organization of the propulsive phase of gait. Hence, one would assume that double support is an important neuromechanical reference during curved walking. For this reason, its duration is increased in mildly affected patients, while its variability is kept within limits. Double-support duration and variability would then degrade to a greater extent later in the degenerative PD process [107]. Interestingly, postural instability problems are dopamine resistant [108], and variability of double support is one of the signs that are refractory to levodopa treatment [27,109,110,111].

### General considerations

Basic gait determinants such as speed and cadence do not exhibit major changes during curved walking in mildly to moderately affected PD patients, walking spontaneously. Hence, curved walking does not represent a necessary challenging barrier for all PD patients [20,112]. This statement must be considered in the light of the specific sample selection. We studied aged healthy adults and high-functioning age-matched non-asymmetric PD patients, who had no obvious walking problems compared to a population of HS exhibiting walking velocities not at the top of the normal range (but with a fully normal gait pattern) [113]. On the one hand, this sample selection restricts the ability to generalize these findings to a general population of PD patients, many of whom present with asymmetry [114]. Interestingly, though, the association between handedness and the side of initial PD symptoms does not seem to affect gait, apart from the obvious effects on dominant-hand use [114]. On the other hand, this emphasizes the significant differences discussed above: the curved trajectory did produce in PD patients shorter stride length, distinct increase of time spent in double support and marked variability of single support duration, in spite of their normal walking speed along linear and curved trajectories.

Possibly, the high variability of gait and the long double-support phase in high-functioning PD patients is connected with their scarce capacity to modulate the stretch responses of the leg muscles [115] or to their attempts to correct the body segmental coordination by continuously readjusting the accuracy of movements [24]. PD patients may adopt additional strategies to perform a complex motor task such as a curvilinear path. As suggested by Guglielmetti et al. [20] and Cheung [116], patients with Parkinson´s disease have to cope with their axial rigidity [117], their poor coordination of intersegmental movements [26,118] and with slower trunk rotation velocities [119,120]. Additionally, PD have to manage the difficulty to generate asymmetric gait pattern and tilted body orientation in order to create a centripetal force in curved paths, while maintaining a good medio-lateral control of the trunk [121]. We did not record here arm-swing presence and amplitude, as in Mirelman et al. [122]. It would not be implausible that the observed abnormalities in the gait phase duration and variability would be partly linked to abnormal arm swing.

### Interaction between posture and gait

The interplay between balance and gait problems in PD is a matter of debate [17,123]. We would note that poor postural control is common in most PD patients, and can interfere with walking [124,125]. In other diseases, walking problems are strongly dependent on impaired balance control, as in peripheral neuropathies [126], stroke [98], cerebellar syndromes [127] and COPD [128]. On the one hand, these links between balance and gait would be compromised in PD during sharp turning [118,129]. On the other, one may note that the limits of stability are reduced in PD [130,131], which would be the counterpart of the reduced forward leaning of the stance leg during the support phase of walking, and lead to shorter steps [132]. A strong predictive relationship between balance deficits and gait impairment has been recently highlighted by Christofoletti et al. [133]. Gait organization *per se* would be instead unimpaired, as inferred from normal muscle activation patterns in the lower extremities during turns in people with PD [134]. Most interestingly, we observed recently in a different cohort of PD patients that walking speed increased after a rehabilitation training exclusively aimed at improving balance [135]. This suggests that better equilibrium control has a positive effect on walking—or that the subtle walking abnormalities exhibited by the present PD patients are a sign of balance, not gait impairment.

### Limitations

One rater scored the UPDRS in our patients. He did not participate in patient recruitment and recording, nor was informed about study procedures. However, Post et al. [136] found considerable rater difference for the whole range of UPDRS scores among different operators. The use of one rater only represents a limit of this study, particularly as far as the asymmetry score is concerned.

No Inertial Measurement Unit (IMU) sensors have been used in this study. IMUs attached to the shoes would have generated additional information about the motion of the feet, in particular when walking on curved paths, such as the use of more steps and smaller turn angles than healthy controls [103,137,138].

Participants were not wearing their own shoes. This might have altered their usual pattern of waking [5]. They wore sneakers of the same type, as in Godi et al. [5], in order to avoid deformation or displacement of the measuring device [139]. Conversely, standardized shoe avoid idiosyncratic effects on spatio-temporal variables of gait and in the duration of limb loading [139,140,141,142,143,144].

The analysis of the statistical power for the differences in the spatio-temporal gait variables between PD and HS showed values higher than 0.80, that is the threshold indicating acceptable and robust results [145] only for curved trajectory. Statistical power was low for the between-group comparisons during the linear trajectory, for all the variables. It is not implausible that minor but significant differences in linear walking might have appeared by comparing larger cohorts.

## Conclusions

The present findings show that curved walking challenges the gait pattern in patients with PD, in whom, at first glance, gait would appear safe and sound and no changes are obvious during linear walking. Since curved walking challenges balance control more than linear walking, and since the operation of the neuronal circuits that shape the activity of the central pattern generator for linear walking in these patients seems to be unspoiled, is it not unlikely that abnormal balance adjustments associated with curved walking are responsible for the observed anomalies.
